# Defining Optimal Soybean Sowing Dates across the US

**DOI:** 10.1038/s41598-019-38971-3

**Published:** 2019-02-26

**Authors:** Spyridon Mourtzinis, James E. Specht, Shawn P. Conley

**Affiliations:** 1 Department of Agronomy, University of Wisconsin-Madison, Madison, WI 53706 USA; 2 Department of Agronomy, University of Nebraska, Lincoln, NE 68583 USA

## Abstract

Global crop demand is expected to increase by 60–110% by 2050. Climate change has already affected crop yields in some countries, and these effects are expected to continue. Identification of weather-related yield-limiting conditions and development of strategies for agricultural adaptation to climate change is essential to mitigate food security concerns. Here we used machine learning on US soybean yield data, collected from cultivar trials conducted in 27 states from 2007 to 2016, to examine crop sensitivity to varying in-season weather conditions. We identified the month-specific negative effect of drought via increased water vapor pressure deficit. Excluding Texas and Mississippi, where later sowing increased yield, sowing 12 days earlier than what was practiced during this decade across the US would have resulted in 10% greater total yield and a cumulative monetary gain of *ca*. US$9 billion. Our data show the substantial nation- and region-specific yield and monetary effects of adjusting sowing timing and highlight the importance of continuously quantifying and adapting to climate change. The magnitude of impact estimated in our study suggest that policy makers (*e.g*., federal crop insurance) and laggards (farmers that are slow to adopt) that fail to acknowledge and adapt to climate change will impact the national food security and economy of the US.

## Introduction

The United States is a major soybean producing country that supplies 34% of global annual soybean production^1^. Most US soybean-producing regions are rainfed, and thus are highly vulnerable to extreme weather events. From 1994 to 2013, variability in growing season precipitation and temperature induced by climate change was estimated to have suppressed soybean seed yield gain *ca*. 30%, effectively a loss of US$11 billion^2^. Drought and elevated air temperatures, now more increasingly frequent due to climate change, are important constraints in crop production across major agricultural areas globally. Thus, the challenge to increase crop yields to meet future demand can be achieved by increasing the rate at which climate change adaptation practices are identified and adopted.

Vapor pressure deficit (Vpd) is a measure of atmospheric water demand with a strong influence on plant transpiratory water loss^3,4^. Increasing Vpd values are generally associated with drought and heat. Improved genetic traits and crop management strategies could help mitigate the projected negative impacts of climate change on crop yields. For example, drought-tolerant traits, introduced through conventional breeding, resulted in soybean transpiration rates that plateaued at Vpd levels above 1.4–2.1 kPa^5–7^. Crop management strategies, such as earlier-than-typical sowing, has also been proposed as a strategy to increase yields in regional studies^8,9^. However, soybean exhibits different sensitivities to weather during varied developmental stages^10^, and therefore, the sensitivity of a crop to climate adaptation strategies and their effectiveness in mitigating drought-induced yield reduction remains unclear.

An important step towards adapting to climate change and mitigating its impact on yield is accurate identification of the weather conditions that most affect crop yield. As has been reported earlier, one option is sowing date adjustment. Regional trials have shown the benefits of earlier sowing^8,9^; however, there is a limit to how much the regional field trials can extrapolate results. Our objective was to examine crop sensitivity to varying in-season weather conditions and to model optimal sowing dates and associated yield and monetary benefits due to sowing date adjustment across the US. To date, there is no similar previous work.

**Figure 1:**
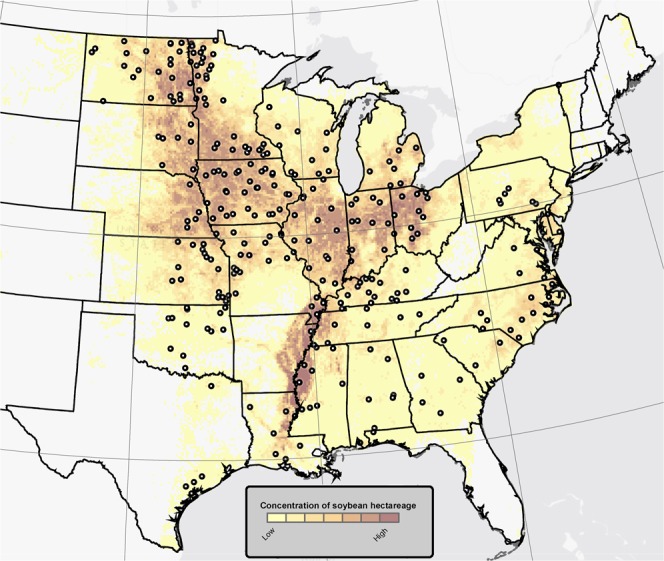
Soybean hectarage distribution in the US. Circles show the locations of the rainfed soybean yield cultivar trials conducted during 2007–2016 in 27 states (n = 1,323 location × year combinations), and the yellow-to-brown coloration denotes relative soybean crop density.

A major obstacle is the lack of an extensive database that includes a wide range of yields and weather conditions. Field-level farmer’s data are an abundant source of information; nevertheless, such data can include unknown confounding factors (*e.g*., unreported and unmanageable field adversities such as hail, frost, flooding), that can significantly affect yield and potentially bias the results. Here, we used data from soybean seed yield cultivar trials performed by agricultural university personnel in 27 states during 2007–2016 (Fig. 1). These multiple-site trials were conducted each year (n = 1,323 location × year yield data) in representative soybean production areas. Within each state, the trial sowing date data (Fig. S1) bracketed the 50% sowing date progress reported by USDA-NASS for each state and year (Fig. S2). These 27 states accounted for ~99% of total US soybean cultivated area (2007 to 2016 average)^11^. The 1,323 location × year yield data were aligned with 1-K-resolution daily weather data. State-wide average yield and weather conditions were calculated resulting in 186 state-year soybean yield and weather condition data.

**Figure 2:**
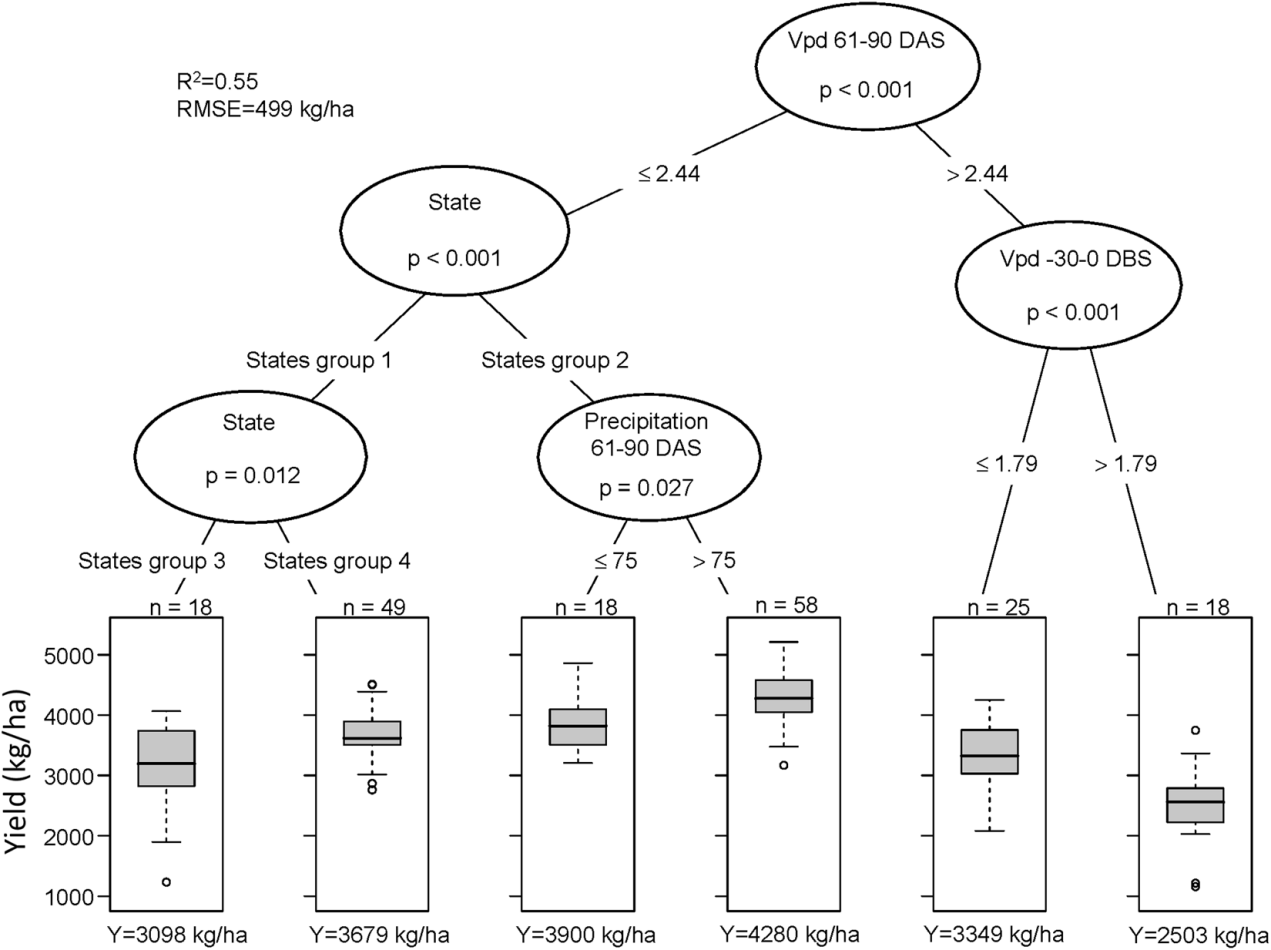
Conditional inference tree for 186 US state-year soybean trial yields (kg/ha) distributed across 27 states during 2007–2016 (Fig. 1). In each boxplot, the central rectangle spans the first to third yield quartiles. The solid line inside the rectangle is the mean which is also numerically shown at the bottom (Y). The number of state-year yields (total = 186) is shown on top of each boxplot (n). The white circles show outlier yields. The acronyms DAS, DBS, and Vpd are, respectively, days after sowing, days before sowing, and vapor pressure deficit, with Vpd reported in kPa and precipitation in mm. States in group 1 include: AL, FL, GA, IA, KS, LA, MN, MO, NC, ND, OK, TN, TX, and VA. States in group 2 include: AR, DE, IL, IN, KY, MI, MS, NE, OH, PA, SD, SC, and WI. States in group 3 include: AL, GA, ND, OK, and TX. States in group 4 include: FL, IA, KS, LA, MN, MO, NC, TN, and VA. A color-coded map of the four groups is shown in Fig. S3.

To identify weather variables during the growing season that had the strongest impact on soybean seed yield in the 10-year, 27-state data set, we used conditional inference regression tree analyses (Fig. 2). Our chosen candidate predictors were: the state in which a trial was performed, cumulative precipitation and solar radiation, average Vpd, maximum temperatures, and relative humidity. We divided the growing season into six successive 30-day time windows from 30 days before sowing (DBS) to 150 days after sowing (DAS), and calculated predictor values for each window, thereby resulting in 31 total candidate weather predictors. The overarching goal of this analysis was interpretation of the model, so additional variables, such as day length, and minimum temperatures, that might enhance the fit of the model, but also might confound with other weather variables, were not included in the analyses.

## Results and Discussion

The conditional inference tree analyses revealed that Vpd during 61 to 90 DAS was the most important predictor of soybean yield (Fig. 2), which was consistent with a finding in a previous study that focused on just three Midwestern states^12^. The lowest trial yields were observed in state-years in which Vpd was greater than 2.44 kPa from 61 to 90 DAS, and Vpd from −30 to 0 DBS was greater than 1.79 kPa. The highest yielding trials were those in which Vpd was lower than 2.44 kPa from 61 to 90 DAS, in 13 states as listed in Fig. 2 legend and colored blue in Fig. S3, and with precipitation greater than 75 mm from 61 to 90 DAS. These results show that the state and amount of precipitation from 61 to 90 DAS are important yield limiting factors mainly in non-drought conditions.

The large yield difference among the levels of Vpd highlights the strong and negative effect of drought in soybean production. Indeed, regression analyses between Vpd and soybean yield indicated that increased Vpd during 61 to 90 DAS reduced yield across the US and 10 years (2007–2016) by 1,135 kg/ha/kPa. This estimated yield loss due to Vpd during 61 to 90 DAS did not vary significantly among states (P > 0.05). The impact of 61 to 90 DAS Vpd >2.44 kPa on yield is exacerbated when coupled with an increased −30 to 0 DBS Vpd, as shown by the regression of latter values on yields. Such harsh growing season conditions resulted in an average soybean yield suppression amounting to 2,074 kg/ha/kPa. This estimated yield loss due to Vpd during −30 to 0 DBS also did not vary significantly among states (P > 0.05).

The sensitivity of soybean yield to variable in-season weather conditions were examined by creating weather datasets that differed from the typical state-specific sowing dates (trial sowing dates set to zero) in 10-day increments (spanning a total of −30 to +30 days) for all states and years in the study. A machine learning model (Fig. S4), calibrated to predict state-year-specific trial soybean yield across the US based on coordinates and weather variables, was applied to estimate yields for each hypothetical sowing date in every state from 2007 through 2016.

**Figure 3:**
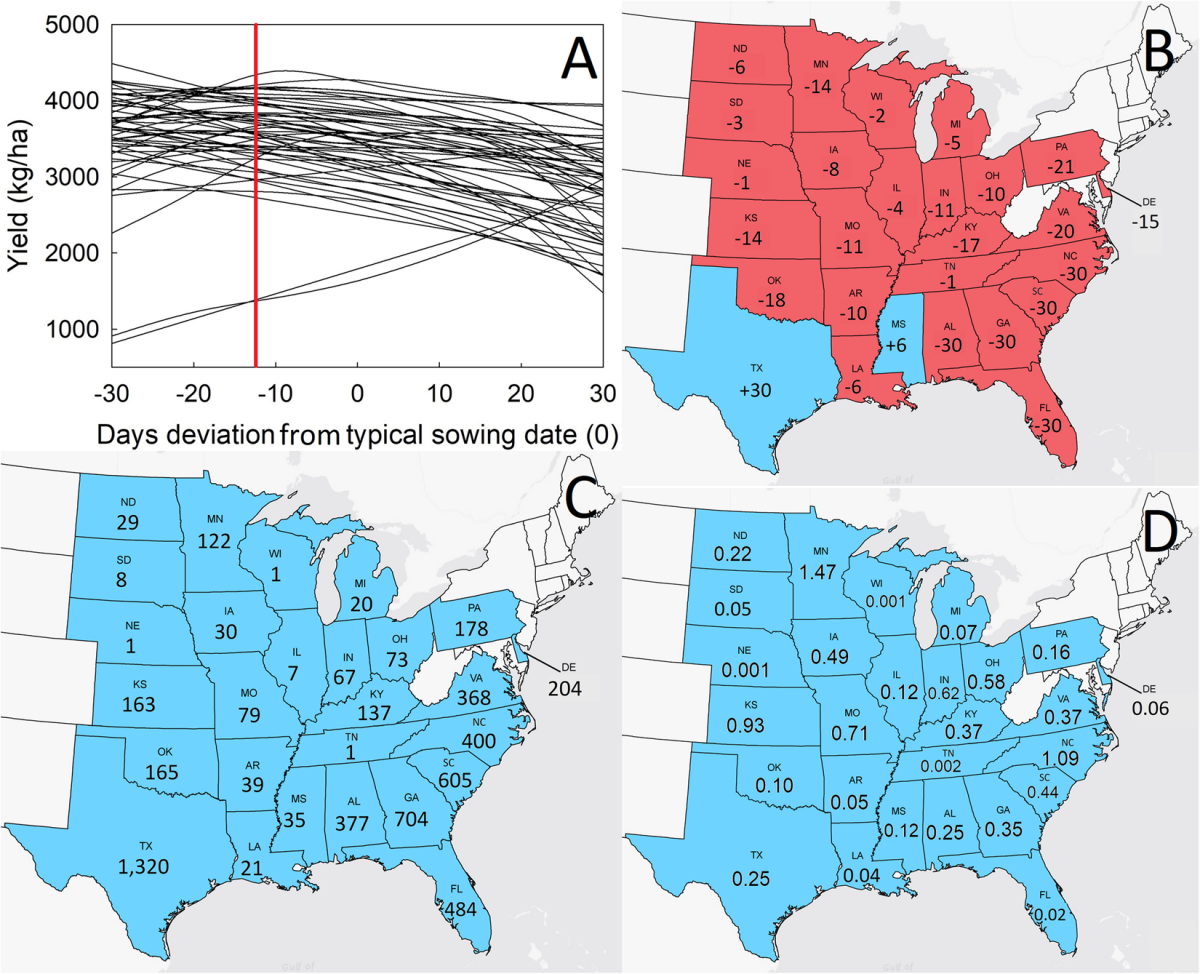
Ten-year average state-specific (n = 27 States) effect (**A**) of sowing date on soybean yield (kg/ha) using weather data sets that differed from the typical sowing date (trials sowing date set to zero) in 10-day increments (spanning a total of −30 to +30 days). The red vertical line shows the US-average predicted optimum sowing date difference from typical. (**B**) Ten-year state-specific optimum sowing date difference from typical. Earlier optimum predicted sowing dates (negative numbers) were identified in red-colored states, but later than typical optimum dates (positive numbers) were identified blue-colored states. (**C**) Simulated 10-year average yield increase (kg/ha) when using the optimum predicted sowing dates in each state. (**D**) Simulated ten-year state-specific cumulative effect of optimum earlier sowing than typical when expressed in terms of soybean producer income (in 2016 inflation-adjusted Billion US$).

A clear trend of increased yields due to earlier sowing was observed within most states (Fig. 3A) across the 10 years of the study. Excluding Texas and Mississippi, where later sowing by 30 and 6 days, respectively, increased yield, sowing 12 days earlier than what was practiced during this decade (2007–2016) across the US would have resulted in a 10% greater total yield. Our results suggest that Southeastern state producers could adjust sowing dates by 30 days earlier than those typically used (Fig. 3B). Such adjustment would have resulted in a 377 to 704 kg/ha average yield increase (Fig. 3C). Mississippi and Texas growers have already adopted an early sowing date strategy *ca*. 1994^13^, and it appears that later sowing by 5 and 30 days, respectively, from what currently is used would have increased yields from 2007 to 2016. In the Midwestern US, soybean producers in states such as Iowa and Ohio could have theoretically experienced a small yield increase (30 and 73 kg/ha, respectively – Fig. 3C) during the past decade by 8 to 10 days earlier sowing, respectively. This result is in agreement with recent regional estimates of early sowing date effect on farmers’ fields^9,14^. In other states with large cultivated areas, such as Nebraska, Illinois, and Wisconsin, producers appear to be already using near optimum sowing dates. It has been reported that earlier sowing dates resulted in a longer sowing-to-first trifoliolate growth stage (V1) period but also advances V1 occurrence on a calendar date basis^15^. This leads to earlier node accrual and floral induction which can optimize the final number of main stem nodes and result in greater yield potential^15^.

In most states, from 2007 to 2016, Vpd from 61 to 90 DAS was constant with delayed sowing, whereas Vpd from −30 to 0 DBS was constant or increasing with later sowing (Fig. S5). The Vpd values, above the critical yield-limiting levels quantified by the conditional tree analysis (Fig. 2), were observed with delayed sowing in most Central and Southern states. In Northern and Midwestern states, although Vpd from 61 to 90 DAS did not exceed the across-US critical levels, delayed sowing would have theoretically resulted in increased Vpd from −30 to 0 DBS and reduced water availability, which would likely resulted in suppressed yields.

Using state-year-specific total income data ($)^11^ and the previously calculated yield change due to sowing date adjustment (Fig. 3C), a 10-year cumulative monetary effect was estimated for each state (Fig. 3D). A substantial monetary gain from earlier sowing was estimated in most soybean producing states. Minnesota, North Carolina, and Kansas would have experienced the greatest monetary gains that could have reached *ca*. US$0.9 to 1.5 Billion. The gains would have been lower in Southern and Southeastern states, despite the greatest yield change due to sowing date adjustment from the Northern states, mainly due to the smaller cultivated area. Indeed, when calculating the monetary effects per hectare, producers in Southeastern states would have benefited the most (Fig. S6). Overall, sowing date adjustment across the continental USA from 2007 through 2016 would have resulted in a cumulative gain of *ca*. US$9 Billion. We note that earlier sowing may be associated with an additional cost for farmers to update or add additional sowing equipment. Still, because such costs can be amortized out over time, we consider our estimates as an upper bound of hypothetical monetary benefits.

**Figure 4:**
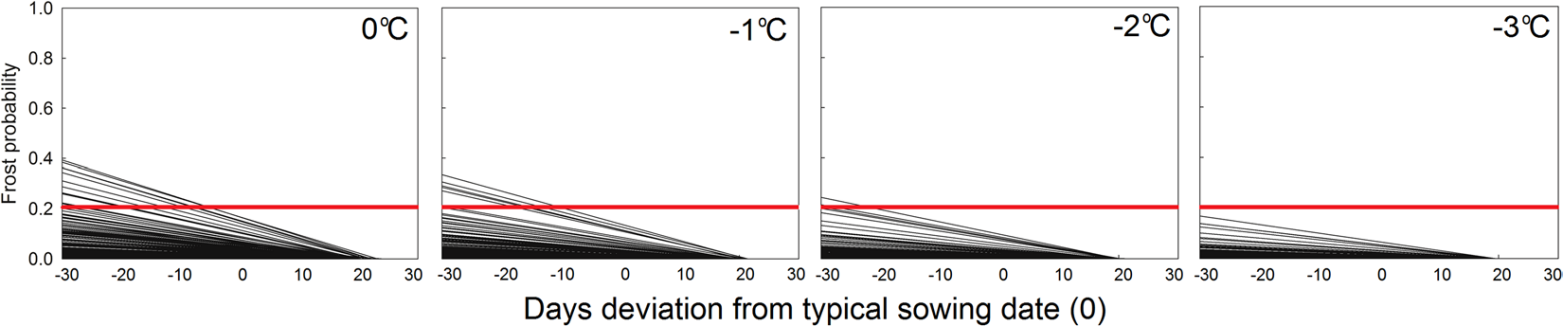
Location-specific (n = 289 locations distributed in 27 states across the US – Fig. 1) spring frost probability for 0, −1, −2, and −3 °C at soybean emergence (at 15 DAS) for 30 DBS to a 30-DAS date bracketing the actual sowing date (set to 0). The red line shows the 20% spring frost probability threshold. The probabilities for each location were calculated using last 46 years of weather data (1981 to 2016).

An important consideration in early sowing is spring frost occurrence, which can damage or destroy the crop (but only after emergence at 15–25 DAS)^16^. The current common recommendation to soybean producers is to sow the first field when frost probabilities are less than 20% on or after emergence. Minor frost damage on emerged seedlings may occur when temperature drops below 0 °C, but becomes more damaging when temperature drops below −2 °C for prolonged periods. In Northern and Midwestern states, where the risk for early frost damage is higher, optimum sowing dates were observed for up to 21 days earlier than what are typically used (Fig. 3B). Using the 21 days earlier sowing as a threshold, 2% of all 289 locations (all in North Dakota, South Dakota, and Minnesota) exceeded the 20% probability threshold for daily minimum temperatures to drop below 0 °C at emergence and only 0.3% had exceeded the 20% probability for daily minimum temperatures to drop below −1 °C (Fig. 4). In the Southeastern states, frost probabilities (Tmin <0 °C) for 30-days earlier sowing was zero. Considering that climate models under different emission scenarios have projected an increase in frost-free season length until the end of the century^17^, these results suggest that frost may not be a serious issue for most regions of the continental US when moving sowing dates earlier into spring.

## Conclusions

Global temperatures are expected to continue increasing until 2100^18^. Furthermore, a 20% cumulative increase in Vpd in July in the Midwest is projected by 2040^12^, driven by increased temperatures and reduced relative humidity. Climate simulations have also estimated up to a 30% reduction in precipitation during summer months in many US regions, including the Midwest and much of the Corn Belt^17^. It is clear that soybean exhibits variable sensitivities to weather during vegetative and reproductive development^10^. To that end, we show here that with state-specific sowing date adjustment, drought impact during sensitive developmental stages could be mitigated.

We acknowledge that more work is needed to extrapolate the results of this work to finer spatial resolution. Previous work has shown that there is significant heterogeneity in soybean sowing dates regionally^19^, and results may change when moving from regional-level to field-level analyses^20^. However, such work would require more locations with better spatial coverage across the US. Additionally, there is a lack of measured daily weather data at appropriate spatial resolution and there are known issues with gridded weather data quality for field-level analysis^21^. In conclusion, currently such data are not existent and to that end, more work is imperative.

Overall, our results agree with previously reported simulated future yield trends (a 7 to 15% increase) due to climate adaptation in wheat, rice, and maize^22^. The results in our study complement the previously measured sensitivity of soybean-related long-term economic returns to regional climatic change^2,23^ by identifying and quantifying climate change-related yield constraints. It is evident that many progressive farmers in the NC US region (e.g., NE and WI), continuously monitor and strive to sow crops earlier on an annual basis. Our results highlight the potential yield and monetary benefit that US farmers can gain due to sowing date adjustment by using the results we report as a point of reference of optimal sowing date in each state. Lastly our findings suggest that the USDA Risk Management Agency should consider updating their antiquated earliest sowing dates for replant payments to reflect current environmental and monetary factors.

## Methods

Soybean yield from non-irrigated cultivar performance trials conducted at sites within each of 27 states from 2007 through 2016 were assembled for this study. Sowing dates in farmer fields were assessed using state-wide publicly available data^11^. The dates when 50% of state-wide hectares were sown every year were interpolated using the data reported in each weekly crop progress report. For all subsequent analysis, yield and sowing dates reported in each trial were used.

Weather data were obtained from the DAYMET^24^ dataset due to its improved accuracy compared to other sources of weather data^21^. Weather variables included were daily minimum and maximum temperatures (Tmin and Tmax, respectively), precipitation, day length, solar radiation, and water vapor pressure. Vapor pressure deficit (Vpd) was estimated as the difference between saturated vapor pressure (0.6107 * exp(17.269 × T/(237.3 + T))) at daily Tmax and at daily Tmin. Relative humidity was calculated by dividing the vapor pressure by the average of saturated vapor pressure at daily Tmax and Tmin.

In every trial, multiple maturity groups (MG) were used. For this study, pooled cultivar yields within the MG that exhibited maximum yield was recorded and used in subsequent analysis under the assumption that the year-specific weather conditions favored this specific MG. Then, for all cultivars in all location × years, sowing date as day of year (doy) was set to zero. The 30-day specific weather conditions, of all the aforementioned weather variables, starting from 30 days before sowing (DBS) up to 150 days after sowing (DAS), were calculated and used as independent variables in subsequent analysis. This allowed the models to capture differential weather sensitivities at different development stages. Finally, the seed yield and weather conditions of all trials within a state × year were averaged to obtain state × year-wide estimates. This resulted in 186 state × year-specific soybean yields.

In our study, weather variables cannot be considered to have been “applied” in a randomized manner, nor replicated with respect to analysis of trial data. Thus, use of traditional linear models (*e.g*., analysis of variance, multiple linear regression) may be misleading and other analysis tools are more appropriate^25^. Consequently, we used the conditional inference regression trees methodology, within the “partykit” package in R (R development Core team, 2016), to identify the 30-day-specific weather variables that affected soybean seed yield across the continental US. For this analysis, state, cumulative precipitation and solar radiation, Vpd, maximum temperatures, and relative humidity were used as independent variables with the ultimate goal to interpret the resulted model. Variables that could confound the results (e.g., Tmin) were not used.

This methodology does not require statistical distribution assumptions, can handle categorical and continuous explanatory variables, is robust to outliers, multicollinearity, heteroscedasticity, and can reveal variable interactions^26^. There is no bias and overfitting issues, and one can estimate a relationship among several variables by binary recursive partitioning in a conditional inference framework^27^. In the first step, the algorithm tests the global null hypothesis of independence between the response variable (*i.e*., yield) and any of the input weather variables. If the hypothesis cannot be rejected, the algorithm stops; otherwise the algorithm selects the input variable with strongest association to the yield response. The association is measured by a p-value which corresponds to an association test for the partial null hypothesis of a single input variable and the response. Then, a binary split is implemented in the selected variable (node) and all steps are recursively repeated. The terminal node accounts for the final subset of yields. The result of this procedure is a tree-like appearance graph where intermediate and terminal nodes are defined according to pre-specified criteria. In our analysis, the criterion for the independence test was based on a Bonferroni p-value of alpha = 0.05. Additionally, to ensure adequate power at all steps, each intermediate node had to account for >37 observations (~20% of total observations), and a terminal node had to consist of >18 observations (~10% of total observations). To avoid overfitting and enhance interpretability, the maximum tree depth was set to 10 nodes. Sensitivity analysis, by allowing the development of larger trees and terminal nodes with a smaller number of observations, did not change the resultant model.

Machine learning regression analysis is a powerful method of predicting continuous responses. The objective of the developed machine learning model was the increased precision of prediction of yet to be observed soybean yields rather than just focusing on an improved fit of the training dataset. Therefore, the data were partitioned into training (85% of all data) and test (15% of all data) datasets, stratified by states within years to ensure representative sampling in both, training and test datasets. The most appropriate model was the one that resulted in the highest R^2^ value and lowest root mean square error in the test dataset.

In this analysis, potential predictors included the aforementioned 30-day-specific and season-wide variables to capture differential weather sensitivities at different development stages and season-wide weather variables. Additionally, to enhance the predictive ability of the model, the number of days with precipitation >1 mm (a), number of days with precipitation <1 mm (b), and the ratio a/b, were calculated for each 30-day period and for the entire season. Finally, coordinates (latitude and longitude), and year were also included in the model to capture non-weather-related trends that could bias estimates of weather effects. For example, a measurable and non-constant effect on yield is the technology adoption yield increase trend over time (year). Omitting this variable would result in potential bias in weather coefficients.

The machine learning model was a functional gradient descent algorithm (boosting) which was developed in R (R development Core team, 2016) utilizing the “mboost” package^28^. After tuning the model, the number of boosting iterations was set to 600 and the step length was set to 0.2. Figure S4 shows the performance of the developed model in fitting the training data (Fig. S4A) and in predicting new data in the validation data set (Fig. S4B). We note that the analysis was repeated using trial level data. However, the performance of the developed model was poor (Fig. S7A,B) presumably due to the inability of gridded weather data to capture weather differences at the trial level^21^. Nevertheless, aggregation of trials to state-level did not alter the observed trends due to variable sowing dates (Fig. S8). Thus, state-wide results are presented due to the superior predictive model when using state-wide *vs*. trial-specific data.

For all locations and years in the study, seven different weather datasets were created by changing sowing date from −30 to +30 days from typical (average) in 10-day increments. The machine learning model was applied then in all weather datasets to simulate soybean yield in each location from 2007 through 2016 for a total of 1,890 simulations. The simulated yields were fitted in a multilevel model to quantify the effect of variable sowing date on soybean yield as has been described elsewhere^29,30^. To assess the state-specific soybean yield variability due to sowing date, a three-level conditional (mixed effect) hierarchical nested linear model was fitted using PROC GLIMMIX in SAS 9.4. The linear and quadratic forms of sowing date were treated as continuous fixed effects, and year, state within year, and sowing date within state within year were considered random effects.

The monetary effect of optimum sowing was calculated in three steps. In the first step, the percentage of yield change due to optimum sowing within each state, as was identified in previous analysis, and the total, state-wide non-irrigated soybean production change (metric tons) in each year (2007 to 2016) were used to estimate the production difference (%). Then, the state-year-specific total income ($)^11^ was adjusted for inflation to 2016 US$ values. Finally, utilizing the previously calculated production change and the state-year-specific inflation adjusted monetary effects, a 10-year cumulative monetary gain was estimated for each state across the US. Additionally, using the 10-year average soybean hectares^11^ in each state, a state-specific average monetary effect, in terms of $/ha, was calculated.

Frost probabilities were calculated using binomial distribution of event occurrence (spring frost *vs*. no frost) for different daily minimum temperature thresholds in all locations of the study (n = 289 locations distributed in 27 states). The last 46 years of weather data (1981 to 2016) for each location were used to calculate the probabilities.

## Supplementary information

SUPPLEMENTARY INFO

## References

[1] Food and Agriculture Organization of the United Nations (FAO), FAO Statistical Databases, http://faostat.fao.org (2017).

[2] Mourtzinis S (2015). Climate-induced reduction in US-wide soybean yields underpinned by region- and in-season specific responses. Nat. Plants.

[3] Ray JD, Gesch RW, Sinclair TR, Allen LH (2002). The effect of vapor pressure deficit on maize transpiration response to a drying soil. Plant Soil.

[4] Lobell DB (2013). The critical role of extreme heat for maize production in the United States. Nat. Clim. Change.

[5] Fletcher AL, Sinclair TR, Allen LH (2007). Transpiration response to vapor pressure deficit in well watered ‘slow wiliting’ and commercial soybean. Environ. Exp. Bot..

[6] Sinclair TR, Zwieniecki MA, Holbrook NM (2008). Low leaf hydraulic conductance associated with drought tolerance in soybean. Physiol. Plant..

[7] Devi JM, Sinclair TR, Chen P, Carter TE (2014). Evaluation of elite southern maturity Soybean breeding lines for drought-tolerant traits. Agron. J..

[8] Rowntree SC (2013). Genetic gain × management interactions in soybean: I. Planting date. Crop Sci..

[9] Rattalino Edreira JI (2017). Assessing causes of yield gaps in agricultural areas with diversity in climate and soils. Agric For Meteorol..

[10] Purcell, L. & Specht, J. E. In Soybeans: Improvement, Production, and Uses 3^rd^ edn (eds Boemma, H. R. & Specht, J. E.) 569–620 (American Society of Agronomy, 2004).

[11] USDA-NASS. 2017. Quick Stats 2.0.USDA-NASS, Washington, DC. www.nass.usda.gov/quickstats/ (accessed April 20 2018).

[12] Lobell DB (2014). Greater sensitivity to drought accompanies maize yield increase in the U.S. Midwest. Science.

[13] Heatherly, L. G. Early soybean production system (ESPS). In Soybean production in the Midsouth; Heatherly, L. G., Hodges, H. F., Eds; CRC Press: Boca Raton, FL, USA, 103–118 (1999).

[14] Mourtzinis S (2018). Sifting and winnowing: analysis of farmer field data for soybean in the US North-Central region. Field Crops Res..

[15] Bastidas AM (2008). Soybean sowing date: The vegetative, reproductive, and agronomic impacts. Crop Sci..

[16] Meyer DW, Badaruddin M (2001). Frost tolerance of ten seedling legume species at four growth stages. Crop Sci..

[17] Walsh, J. *et al*. Ch. 2: Our changing climate. Climate change impacts in the United States: The third national climate assessment, J. M. Melillo, Terese (T. C.) Richmond, and G. W. Yohe, Eds, U.S. Global change research program, 19–67 (2014).

[18] IPCC Climate Change 2013: The Physical Science Basis (eds Stocker, T. F. *et al*.) (Cambridge Univ. Press, 2014).

[19] Urban D, Guan K, Jain M (2018). Estimating sowing dates from satellite data over the U.S. Midwest: a comparison of multiple sensors and metrics. Remote Sens. Environ..

[20] Jain M (2017). Using satellite data to identify the causes of and potential solutions for yield gaps in india’s wheat belt. Environ. Res. Lett..

[21] Mourtzinis S, Rattalino Edreira JI, Conley SP, Grassini P (2017). From grid to field: Assessing quality of gridded weather data for agricultural applications. Eur. J. Agron..

[22] Challinor AJ (2014). A meta-analysis of crop yield under climate change and adaptation. Nat. clim. change.

[23] Specht JE, Hume DJ, Kumudini SV (1999). Soybean Yield Potential-A Genetic and Physiological Perspective. Crop sci..

[24] Thornton, P. E. *et al*. Daymet: Daily Surface Weather Data on a 1-km Grid for North America, Version 3. ORNL DAAC, Oak Ridge, Tennessee, USA (2017).

[25] Ludwig DA (2005). Use and misuse of p-values in designed and observational studies: guide for researchers and reviewers. Aviat. Space Environ. Med..

[26] Tittonel P, Shepherd KD, Vanlauwe B, Giller KE (2008). Unravelling the effects of soil and crop management on maize productivity in smallholder agricultural systems of western Kenya-An application of classification and regression tree analysis. Agric. Ecosyst. Environ..

[27] Hothorn T, Hornick K, Zeileis A (2006). Unbiased recursive partitioning: A conditional inference framework. J. Comput. Graph. Stat..

[28] Hofner B, Mayr A, Robinzono N, Schmid M (2014). Model-based boosting in R-A hands-on tutorial using the R package mboost. Comput. Stat..

[29] Mourtzinis S (2018). Soybean response to nitrogen application across the United States: A synthesis-analysis. Field Crops Res..

[30] Bell, A. B., Ene, M., Smiley, W. & Scoeneberger, J.A. A multilevel model primer using SAS® PROC MIXED. SAS global forum. Statistics and data analysis. Paper 433, (http://support.sas.com/resources/papers/proceedings13/433-2013.pdf) (accessed April 20 2018) (2013).

