# Supplementary material for: Defining Optimal Soybean Sowing Dates across the US
Source: Sci Rep. 2019 Feb 26;9:2800. doi: 10.1038/s41598-019-38971-3 (PMC6391372; doi:10.1038/s41598-019-38971-3)
Supplement: Supplementary file 1 — SUPPLEMENTARY INFO [file 41598_2019_38971_MOESM1_ESM.docx]

**Defining Optimal Soybean Sowing Dates across the US**

Spyridon Mourtzinis^1*^, James E. Specht^2^, and Shawn P. Conley^1^

^1^Department of Agronomy, University of Wisconsin-Madison, Madison WI 53706.

^2^Department of Agronomy, University of Nebraska, Lincoln NE 68583.

^*^Correspondence to [agstat001@gmail.com](mailto:agstat001@gmail.com)


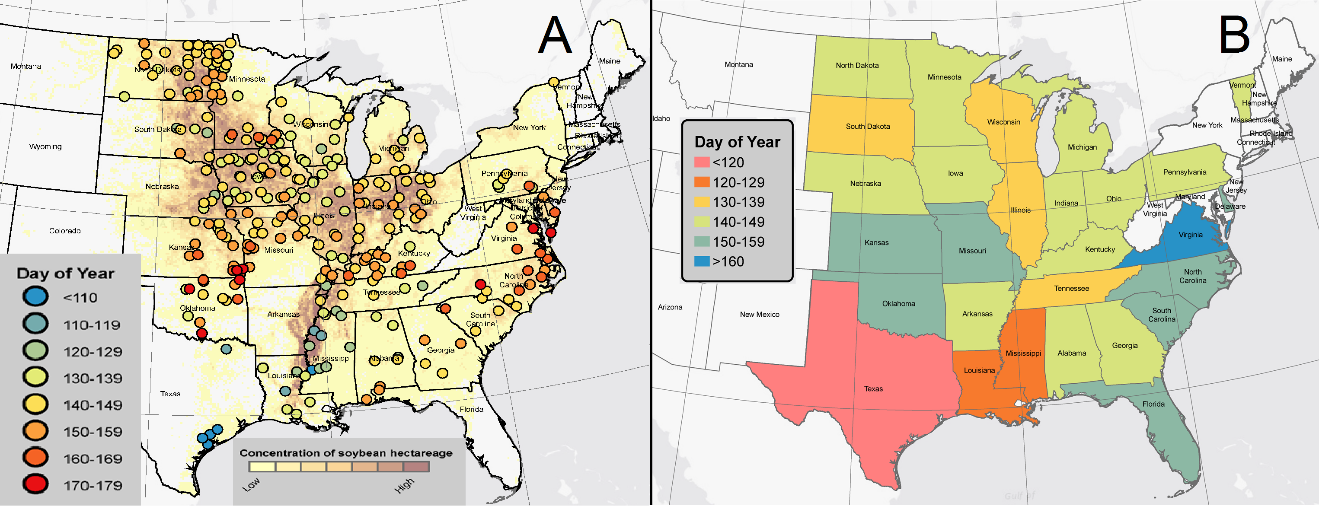


Fig. S1. Location-specific (A) and state-wide (B) average cultivar trial sowing dates expressed as day of year (DOY) across the US from 2007 through 2016.


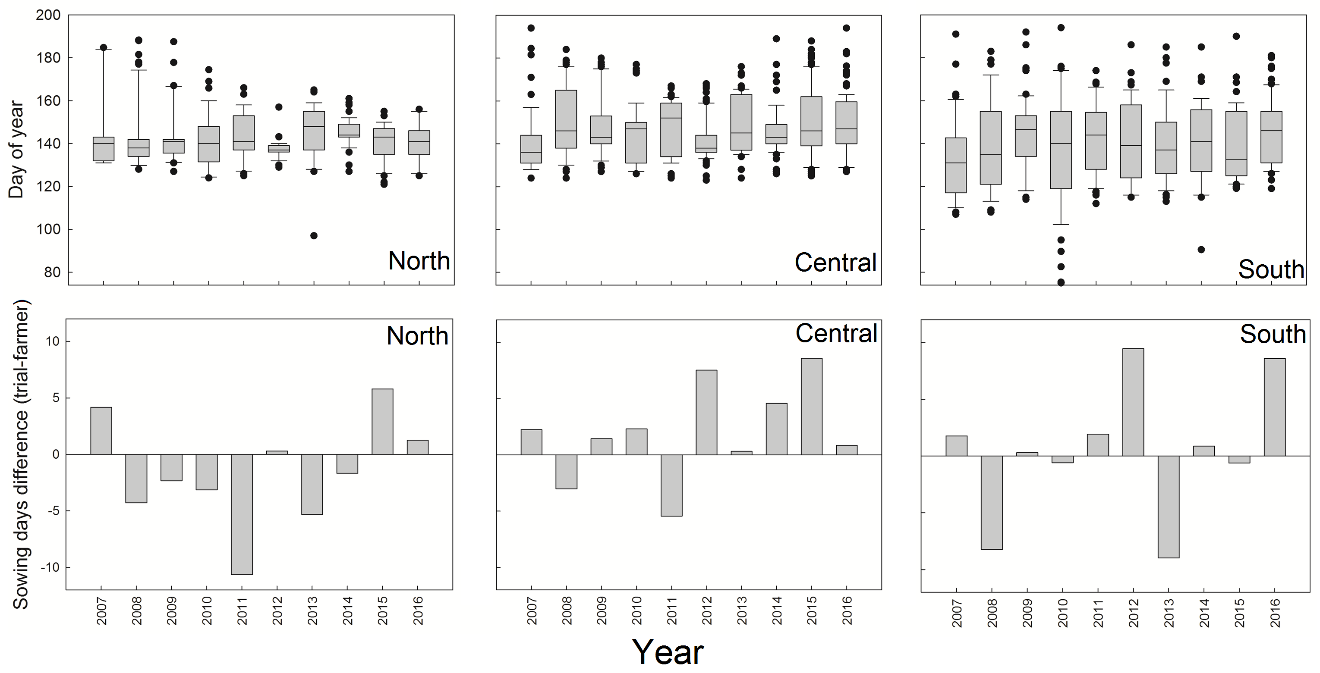


**Fig. S2. The box plots show the distribution of trial sowing dates from 2007-2016 across northern (North Dakota, South Dakota, Wisconsin, Minnesota, and Michigan), central (Nebraska, Kansas, Iowa, Illinois, Missouri, Indiana, Ohio, Kentucky, Pennsylvania, Virginia, and Delaware) and southern (Oklahoma, Texas, Arkansas, Mississippi, Louisiana, Tennessee, Alabama, Georgia, Florida, North and South Carolina) US.** The bar plots show the year-specific comparison of trial sowing dates *vs*. dates of 50% farmer completion of sowing on a region-wide basis.


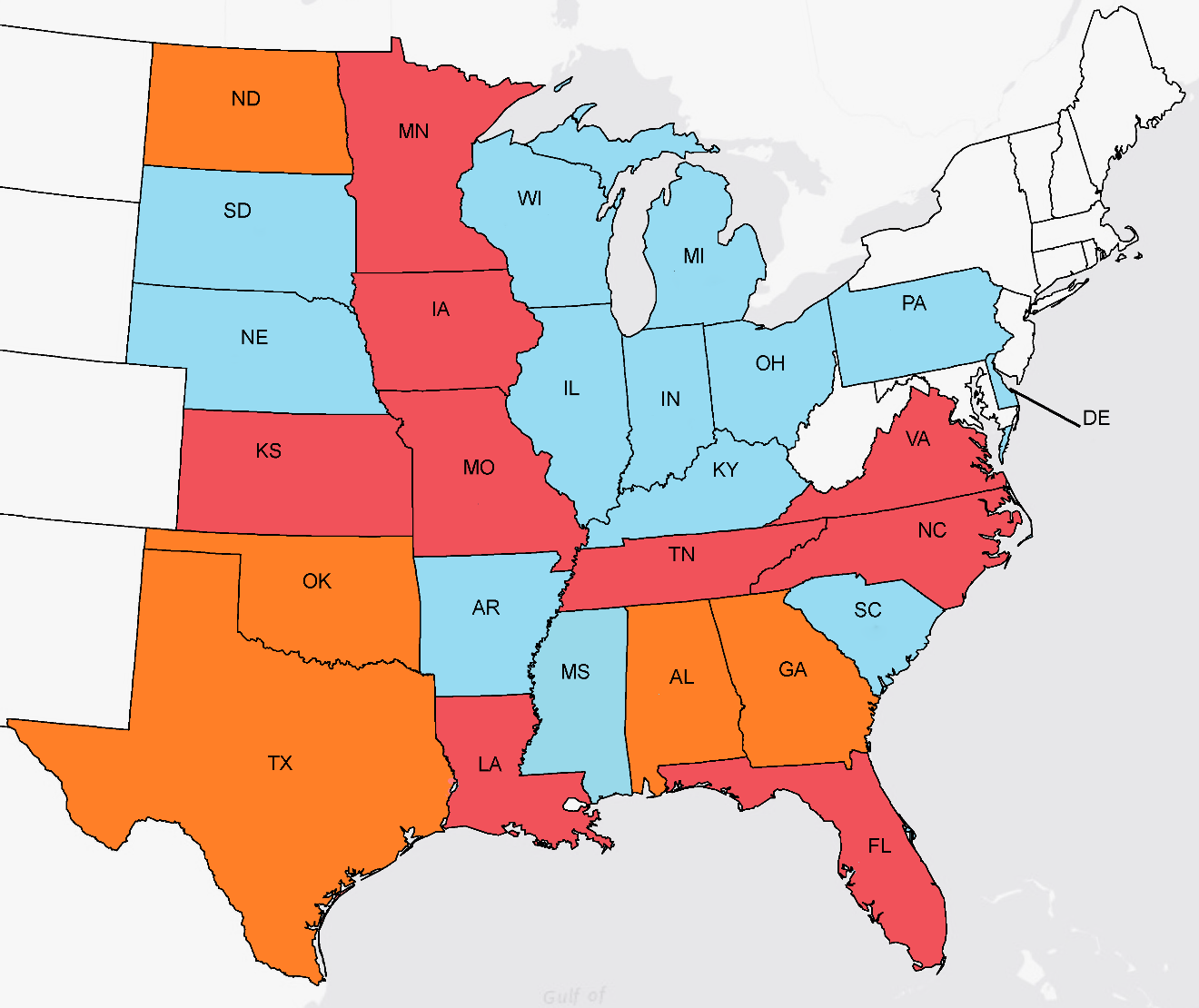


**Fig. S3**. **Groups of states as were delineated in the conditional inference tree analysis in Fig 2. States in group 1 have orange and red color, states in group 2 have blue color, states in group 3 have orange color, and states in group 4 have red color.**


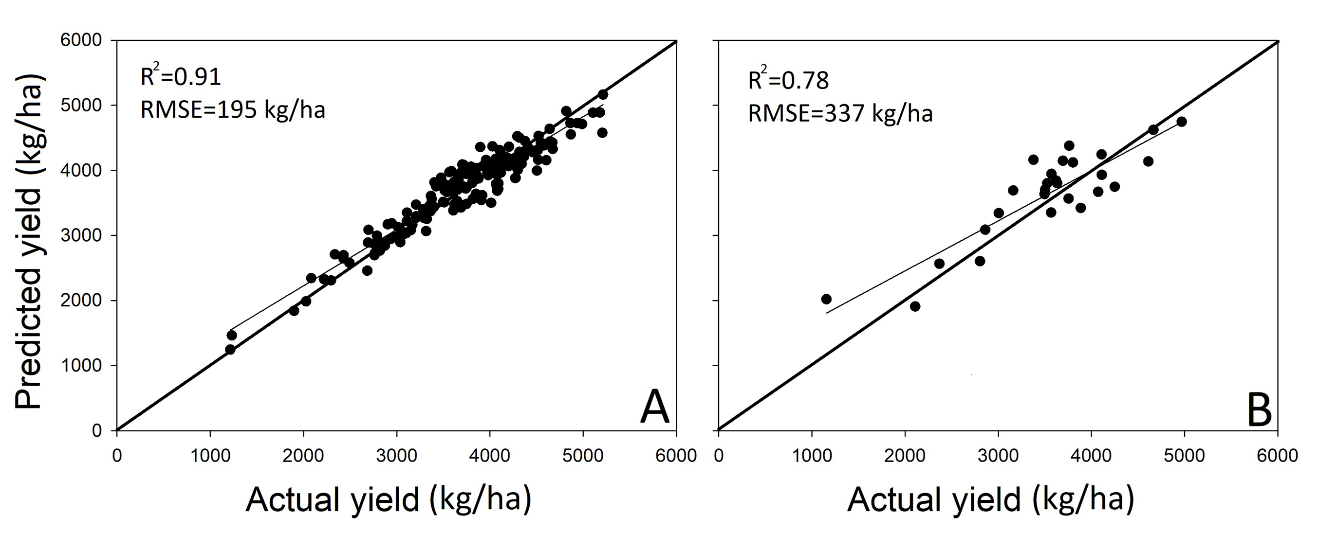


Fig. S4. Comparison of actual and predicted yields in the training (A) and validation (B) data set from the machine learning regression model. Note: R^2^=coefficient of determination, and RMSE=root mean square error.


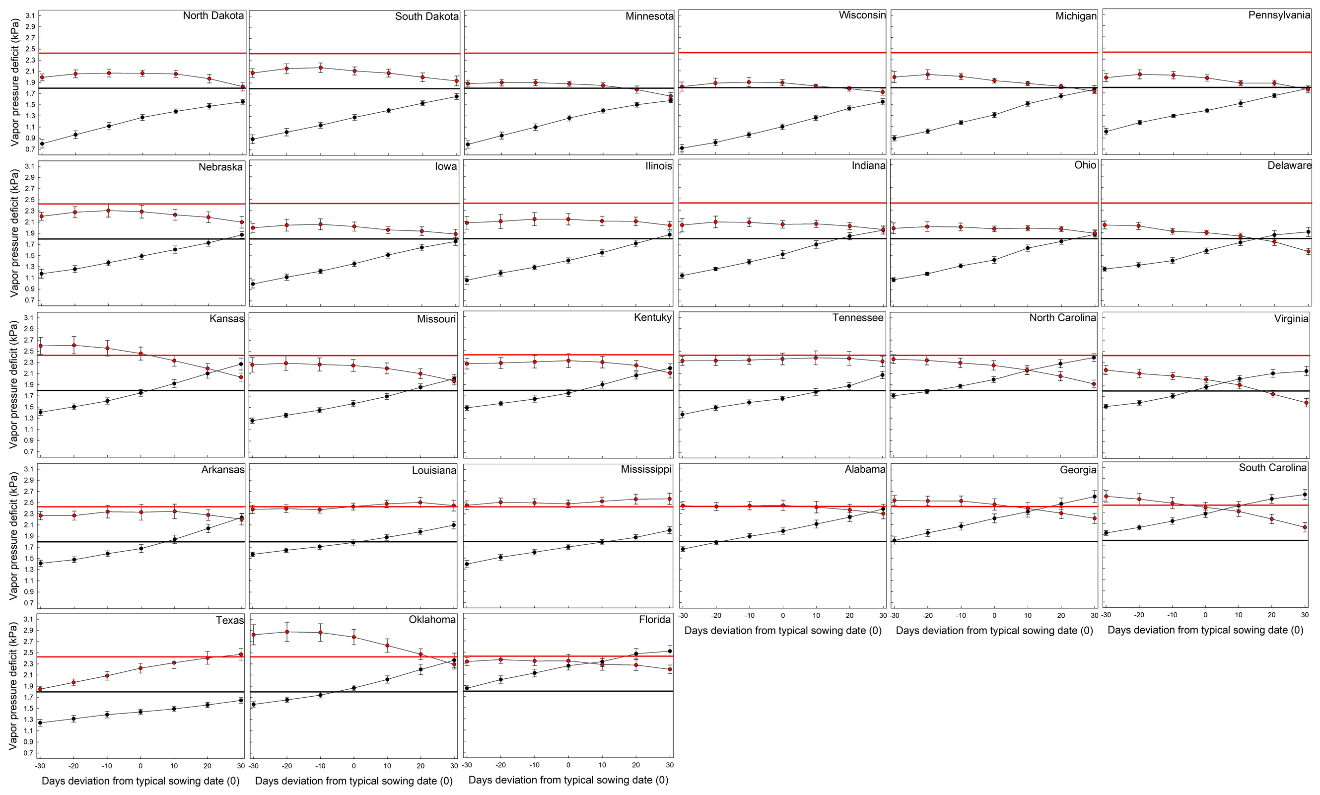


**Fig. S5**. **State-specific (n=27 states) vapor pressure deficit trend for the years 2007-2017 using weather data sets that differed from the typical sowing date (trials sowing date set to zero) in 10-day increments (spanning a total of -30 to +30 days) to show the impact of a 30-day sowing deviation on VPD.** Red solid circles show the vapor pressure deficit (Vpd) during 61 to 90 days after sowing (DAS) whereas, black circles show the Vpd during -30 to 0 days before sowing (DBS). The red and black horizontal lines show the critical yield-limiting Vpd values (2.44 and 1.79 kPa, respectively) during 61 to 90 and -30 to 0 DAS and DBS respectively, as were estimated in the conditional inference tree analysis (Fig. 2), respectively. The bi-directional standard errors show the standard errors of the Vpd mean for each date.


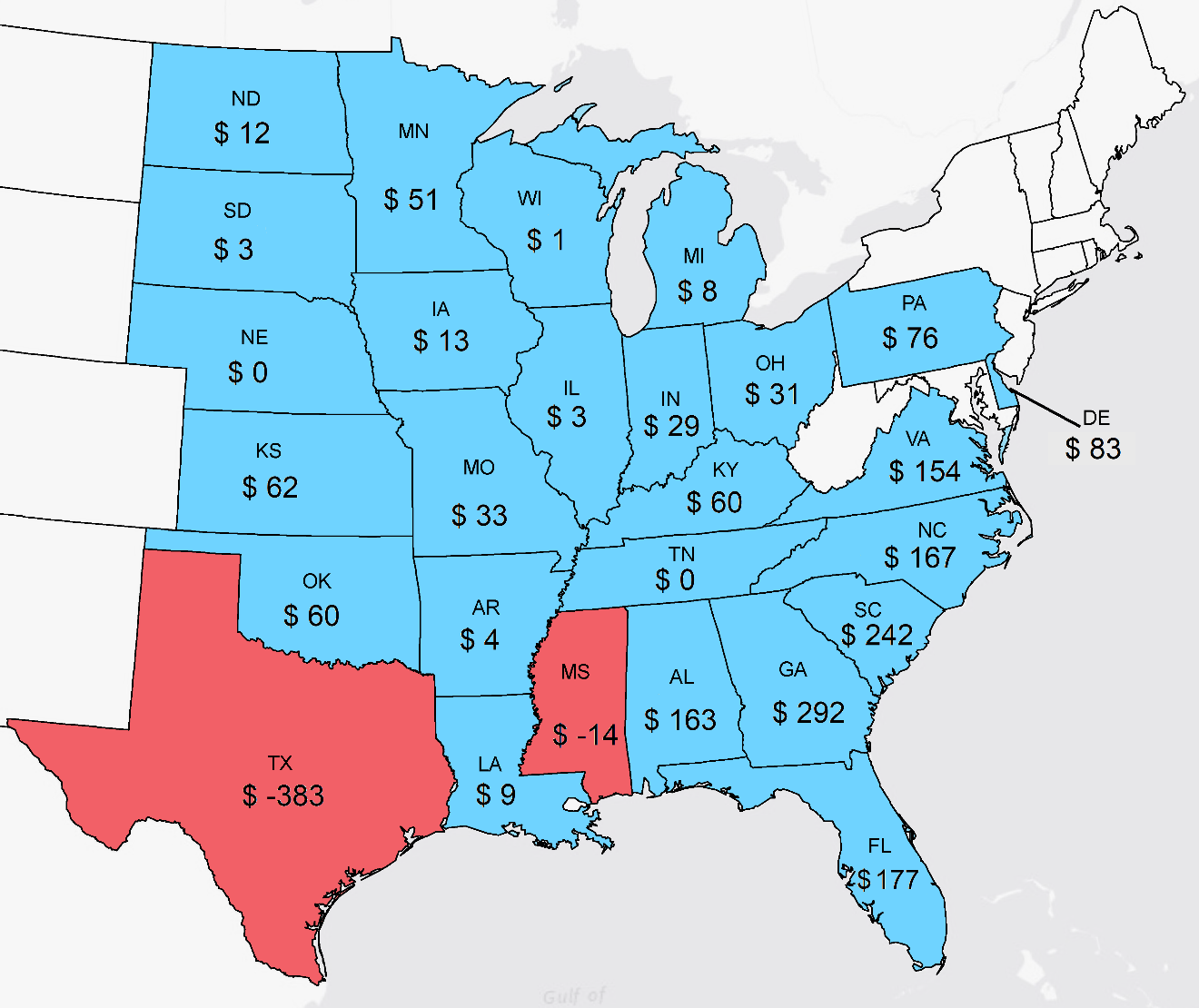


**Fig. S6**. **Ten-year average state-specific (n=27 states) monetary effect when using the optimum identified sowing dates in each state (expressed in 2016 inflation-adjusted US$ per hectare).**


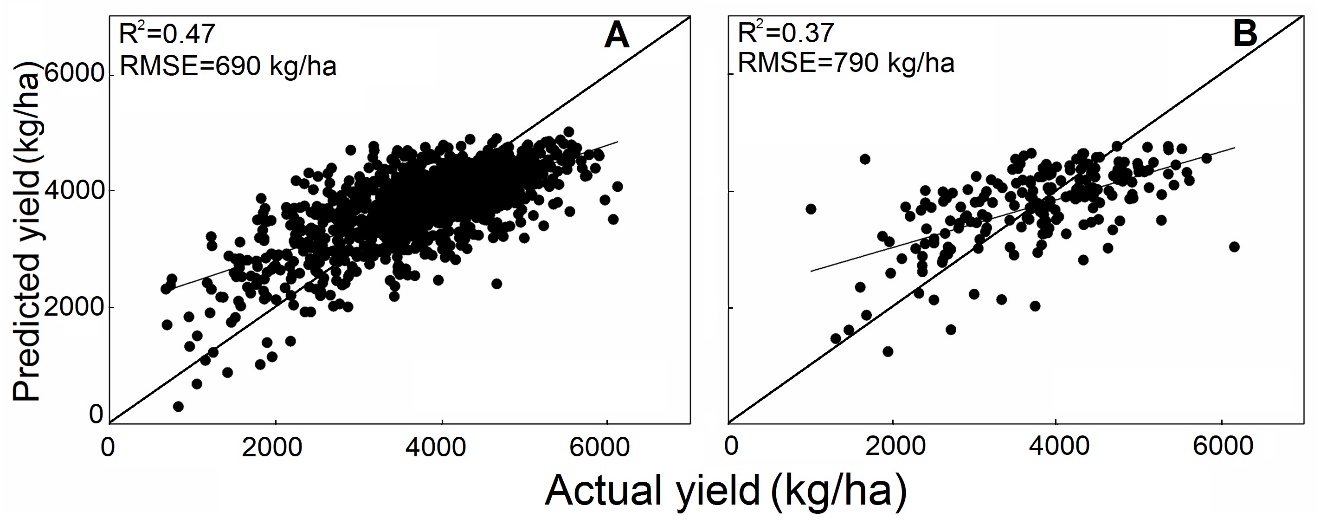


**Fig. S7.** **Comparison of actual and predicted yields in the training (A) and validation (B) data set using trial-level data.** Note: R^2^=coefficient of determination, and RMSE=root mean square error.


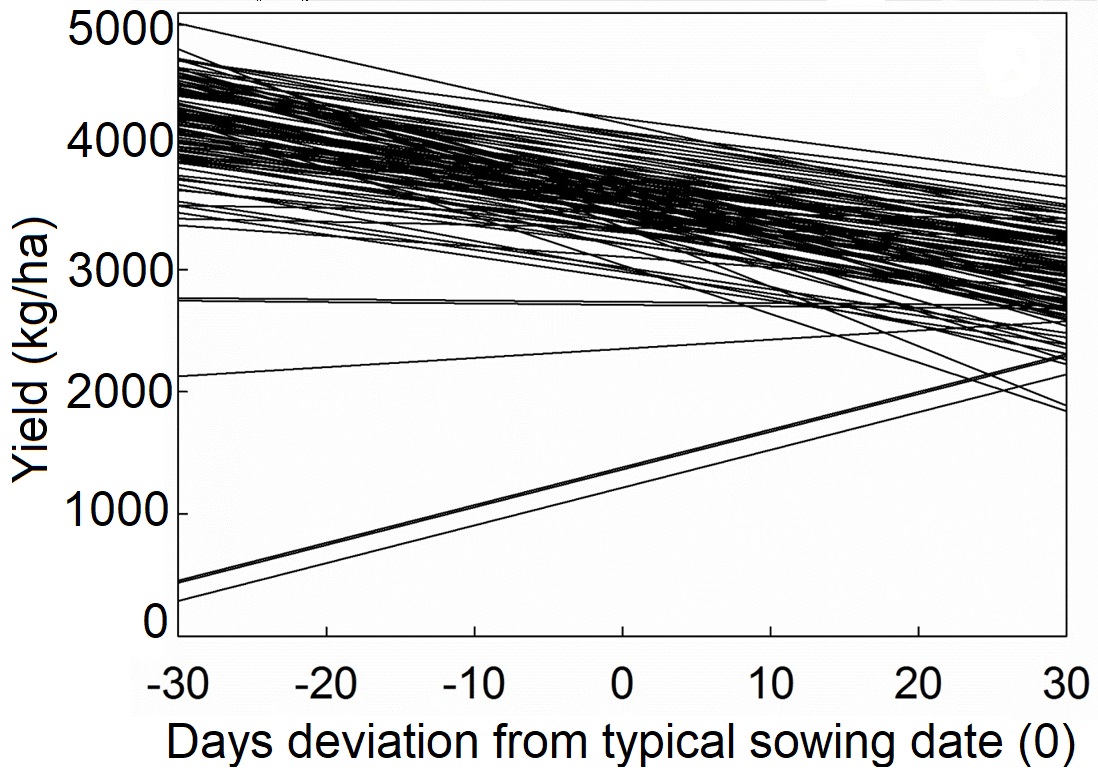


**Fig. S8.** **Trial-specific average effect of sowing date on soybean yield (kg/ha) using weather data sets that differed from the actual sowing date (set to zero) in 10-day increments (spanning a total of -30 to +30 days).**
